# Surfactants as Microbicides and Contraceptive Agents: A Systematic *In Vitro* Study

**DOI:** 10.1371/journal.pone.0002913

**Published:** 2008-08-06

**Authors:** Otilia V. Vieira, Diego O. Hartmann, Carla M. P. Cardoso, Daniel Oberdoerfer, Marta Baptista, Manuel A. S. Santos, Luis Almeida, João Ramalho-Santos, Winchil L. C. Vaz

**Affiliations:** 1 Centre for Neuroscience and Cell Biology, University of Coimbra, Coimbra, Portugal; 2 Department of Biology and CESAM, University of Aveiro, Aveiro, Portugal; 3 Faculty of Pharmacy, University of Coimbra, Coimbra, Portugal; 4 Department of Zoology, University of Coimbra, Coimbra, Portugal; 5 Department of Maternal-Fetal Medicine, Genetics and Human Reproduction, University Hospitals of Coimbra, Coimbra, Portugal; 6 Department of Chemistry, University of Coimbra, Coimbra, Portugal; Yonsei University, Republic of Korea

## Abstract

**Background:**

The urgent need for cheap and easy-to-use protection against both unwanted pregnancies and sexually transmitted diseases has stimulated considerable interest in the use of surfactants as microbicides, anti-viral, and contraceptive agents in recent years. In the present study we report a systematic *in vitro* evaluation of the microbicidal, anti-viral and contraceptive potential of cationic, anionic, zwitterionic, and non-ionic surfactants.

**Methodology/Principal Findings:**

Toxicity was evaluated in mammalian columnar epithelial (MDCK) cells, human sperm cells, *Candida albicans*, *Escherichia coli*, *Pseudomonas aeruginosa*, *Neisseria gonorrhoeae*, *Streptococcus agalactiae* and *Enterococcus faecalis*. The inhibition of adenovirus and lentivirus infection of MDCK cells was also tested. A homologous series of cationic surfactants, alkyl-N,N,N-trimethylammonium bromides (C_n_TAB), with varying alkyl chains were shown to be bactericidal and fungicidal at doses that were related to the surfactant critical micelle concentrations (CMC), all of them at concentrations significantly below the CMC. In general, bacteria were more susceptible to this surfactant group than *C. albicans* and this organism, in turn, was more susceptible than MDCK cells. This suggests that the C_n_TAB may be useful as vaginal disinfectants only in so far as bacterial and fungal infections are concerned. None of the surfactants examined, including those that have been used in pre-clinical studies, showed inhibition of adenovirus or lentivirus infection of MDCK cells or spermicidal activity at doses that were sub-toxic to MDCK cells.

**Conclusions/Significance:**

The results of this study lead us to propose that systematic analysis of surfactant toxicity, such as we report in the present work, be made a mandatory pre-condition for the use of these substances in pre-clinical animal and/or human studies.

## Introduction

The treatment and prevention of sexually transmitted diseases (STDs) is a growing challenge since more and more pathogens are developing multi-drug resistance and effective vaccines do not exist for the majority of them. On the other hand, the world population continues to grow at an alarming rate with a high incidence of unwanted pregnancies. Condoms, when used correctly and consistently, provide a high level of protection against STDs (including HIV) and pregnancies, but many women lack the social and/or economic power to persuade their partners to use them. These facts have highlighted the importance of efforts to develop other approaches to prevent STDs and unplanned pregnancies, especially women-targeted methodologies such as vaginal prophylactic products, especially microbicides [1]. Nonoxynol-9 (N-9), a non-ionic surfactant that is widely used as an over-the counter spermicide was tested as a vaginal prophylactic against HIV infection [2], [3] and, although this surfactant provides protection against some STDs and, *in vitro*, destroys HIV [4], Phase III studies showed that N-9 tends to irritate vaginal mucosa and facilitate HIV transmission [5], [6] and that vaginal irritation increases with frequency of use and dose [7]. More recently Phase III studies have shown that C31G, a mixture of a zwitterionic and a non-ionic surfactant, in a formulation known as “SAVVY Vaginal Gel” is also not useful in prevention of HIV infection in humans [8], [9]. The failure of these Phase-III studies have created a sense of urgency to accelerate the pace of research in this area and to increase efforts to study compounds, particularly those that even at high concentrations, appear to have no effect on the structural integrity and function of the cervico-vaginal or rectal epithelium. Several candidate microbicides such as vaginal acid-buffering agents that maintain a protective vaginal pH, antiretroviral drugs specific for HIV, inhibitors of viral entry into animal cells and detergents or surfactants have been proposed and some of them are being tested in pre-clinical studies [1]. Because of their amphiphilic nature, surfactants are generally believed to act at the level of the cell membrane. Differences between the chemical and physical properties of the pathogen and the host membrane as well as the manner in which the membranes are related to the cell physiology could be profitably exploited. In view of their potential in this regard we attempted to answer the following questions: Are surfactants really useful as vaginal microbicides and contraceptives? Are there good laboratory models to test their utility in this regard?

The answers to these questions require a multi-step, systematic investigation on the effect of different types of surfactants on fungal and bacterial growth, viral infection and the viability of mammalian cells (particularly polarized epithelial cells) and human sperm cells before any possible application is proposed. The results have also to be related to the physico-chemical properties of the surfactants since these are known to condition the effect of surfactants on lipid bilayer membranes [10]–[12]. Therefore, as a first step, we tested anionic (sodium dodecylsulfate, SDS), cationic (a homologous series of alkyl-N,N,N-trimethyammonium compounds with varying alkyl chain), zwitterionic (N-dodecyl-N,N-dimethylammonium-propanesulfonate, DDPS) and nonionic (Triton X-100 and Nonoxynol-9) detergents. These surfactants are all commercially available. SDS, known as an antimicrobial agent able to inactivate HIV in vitro [4], [13], is widely used in personal hygiene (e.g. toothpaste) and cleaning products. C_n_TAB are also known as microbicides and antifungal agents [14]. Nonoxynol-9 is a commonly used spermicide and lubricant in condoms and its structural homolog, Triton X-100 was studied because its action on lipid bilayers and cell membranes is well documented. Since surfactants are amphiphilic molecules, the first locus of their interaction with cells of any kind is the cell membrane and this interaction is related to their partition coefficient between the aqueous and membrane phases which, in its turn, is related to the Critical Micelle Concentration (CMC) of the surfactant. Therefore, another novelty of this study is that surfactant effects were compared taking into account their CMC.

We show here that only the C_n_TAB were bactericidal and fungicidal at concentrations that were not toxic to mammalian columnar epithelial cells grown to confluence. At concentrations that were sub-toxic to the mammalian epithelial cells all the surfactants examined were neither spermicidal nor did they prevent viral infection of the epithelial cells.

## Results

### Bactericidal properties of surfactants

We chose *Escherichia coli*, *Pseudomonas aeruginosa*, *Neisseria gonorrhoeae*, *Streptococcus agalactiae*, and *Enterococcus faecalis* as bacterial models. The effect of four surfactant classes with similar apolar parts; namely, cationic (C_12_TAB), anionic (SDS), zwitterionic (DDPS), and nonionic (Triton X-100) were first tested for their effects on the growth curves of *E. coli* at concentrations corresponding to various fractions and/or multiples of their respective CMCs. The CMC of the surfactants was used as a reference concentration for the following reason: The formation of surfactant micelles in aqueous media may be considered, under certain conditions, to be similar to a phase separation between an aqueous solution of surfactant monomers and a micellar phase. The surfactant concentration at which this phase separation occurs is the CMC. Within a given homologous series of surfactants the CMC is linearly proportional to the free energy of partitioning of the apolar part of the surfactant between an apolar environment such as a micelle or the lipid bilayer of a cell membrane and the aqueous phase [15]. It follows, then, that the toxic effects of any surfactant homologous series must be compared with the CMC as the reference concentration. Even if the surfactants act at some intracellular level, they must cross the cell membrane in order to do so and, again, their partitioning into the apolar part of the membrane becomes a critical step. A relationship between surfactant toxicity and CMC was pointed out by Ahlström and co-workers [16] who attributed the phenomenon to the association of pre-micellar complexes with the membranes. We propose that the relationship between toxicity and CMC is simply a result of the thermodynamics of partitioning of amphiphiles between the aqueous phase and an apolar environment, be it a micelle or a membrane.

**Figure 1:**
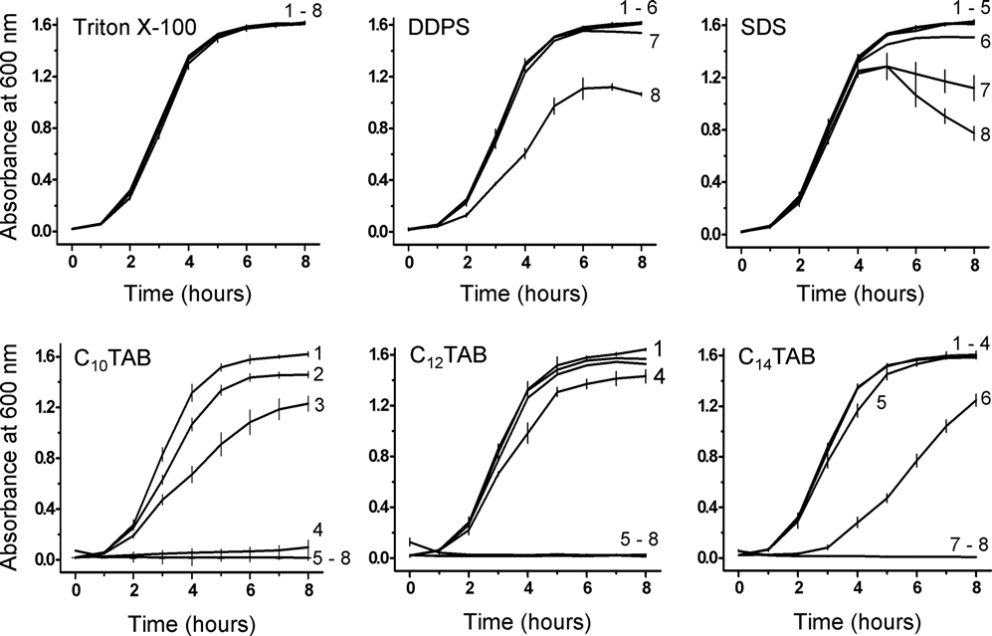
Effect of surfactants on the growth of *Escherichia coli* (wild type). Bacterial growth was assessed by measuring the absorbance at 600 nm as a function of time, in the presence or absence (control) of detergents. Surfactants were added at time zero (lag-phase growing E. coli). Concentrations of the surfactants are given relative to their respective CMC. For convenience the CMC of the detergents used are: Triton X-100, 2×10^−4^ M; DDPS, 2×10^−3^ M; SDS, 2.6×10^−3^ M; C_10_TAB, 4.0×10^−2^ M; C_12_TAB, 3.5×10^−3^ M; and C_14_TAB, 2.8 × 10^−4^ M [24]. For all surfactants, the following concentrations were used: 1) CMC×0 (control); 2) CMC/100; 3) CMC/50; 4) CMC/25; 5) CMC/10; 6) CMC/5; 7) CMC/2; and 8) CMC/1. The results shown are the mean of three independent experiments. Standard deviations are only shown for the control and for those curves in which there is a noticeable but not complete inhibition of growth, these curves are identified by the number that corresponds to the respective surfactant concentration. Overlapping curves are not individually identified.

As shown in Figure 1, C_12_TAB began inhibiting the growth of *E. coli* at a concentration of CMC/25 and was totally inhibitory at all higher concentrations examined. DDPS showed some inhibition of bacterial growth only at CMC/1 and SDS at a concentration of CMC/2. The non-ionic surfactant, Triton X-100, did not inhibit the bacterial growth even at concentrations as high as CMC×100 (not shown). For convenience the CMC of these detergents is listed in the legend to Figure 1.

Given the results obtained with C_12_TAB, we examined the effect of some homologues of this surfactant, namely C_10_TAB and C_14_TAB, on the growth curves of *E. coli*. The results are also shown in Figure 1. Growth of the bacteria was inhibited by all three C_n_TAB homologues albeit at different concentrations relative to their respective CMC. The toxicity of C_10_TAB began to be noticeable at CMC/100, that of C_12_TAB at CMC/25 and that of C_14_TAB at CMC/5. The bromide anion was not the determinant in the toxicity of these surfactants since no differences were found between the toxicities of the C_n_TAB and their chloride analogues (results not shown).

**Figure 2:**
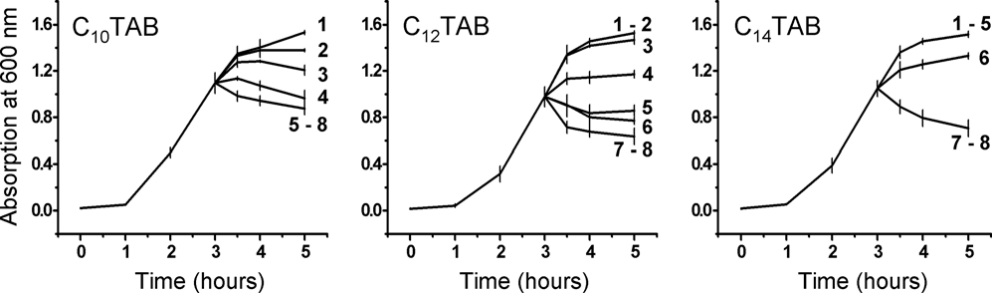
Effect of the three C_n_TAB homologues when incubated with log-phase growing *E. coli*. Concentrations are given relative to the CMC of each surfactant. Other details are as in Figure 1.

The effect of the C_n_TAB surfactants was also tested on *E. coli* cultures growing at log-phase. The results are shown in Figure 2 and Table 1. When inhibition of bacterial growth was seen, the effect was bactericidal since plating the cells on LB/agar plates following removal of the surfactant by centrifugation and washing of the cells (Table 1) did not result in the formation of bacterial colonies. C_12_TAB and C_14_TAB show no or only a very small bactericidal effect at their respective CMC/100 concentrations. In contrast, C_10_TAB was still effective at its CMC/100 concentration and significant bactericidal activity was seen at 60 and 120 min of exposure at the concentration of CMC/100 (Table 1).

**Table 1:** Bactericidal Activity of C_n_TAB Surfactants.

Surfactant	Concentration	Number of Colonies (% of Control) after Exposure Times of		
		30 min	60 min	120 min
C_10_TAB	CMC/100	92.8±12.4	65.0±10.7	54.1±7.8 ***
	CMC/50	73.6±4.3	12.3±0.4 ***	12.3±1.5 ***
	CMC/25	18.9±9.5 ***	2.6±1.7 ***	0.9±1.6 ***
	CMC/10	12.5±3.9 ***	0.4±0.3 ***	0 ***
	CMC/5	0.3±0.5 ***	0 ***	0 ***
	CMC/2	0 ***	0 ***	0 ***
	CMC/1	0 ***	0 ***	0 ***
C_12_TAB	CMC/100	90.1±4.6	79.0±3.9	76.0±3.2
	CMC/50	77.1±2.3 ***	37.0±1.3 ***	27±4.0 ***
	CMC/25	37.3±5.7 ***	10.2±0.2 ***	8.3±1.5 ***
	CMC/10	14.6±2.5 ***	11.0±3.3 ***	6.1±0.2 ***
	CMC/5	1.1±1.9 ***	2.6±3.7 ***	1.0±0.1 ***
	CMC/2	0.4±0.7 ***	0.7±1.2 ***	1.2±2.2 ***
	CMC/1	0.4±0.5 ***	0 ***	0 ***
C_14_TAB	CMC/100	98.9±3.8	92.3±3.2	92.0±6.9
	CMC/50	89.4±1.2	73.5±8.3	74.4±6.4
	CMC/25	87.4±10.9	59.0±4.0	54.8±8.4 ***
	CMC/10	57.6±4.0 ***	54.3±8.7 ***	46.0±3.8 ***
	CMC/5	30.9±3.7 ***	22.7±2.3 ***	18.3±1.6 ***
	CMC/2	0.5±0.7 ***	0.7±0.6 ***	1.0±1.0 ***
	CMC/1	0 ***	0 ***	0 ***

Cultures of *Escherichia coli* were made up to the stipulated concentrations of the surfactants and gently swirled for the stipulated times after which the cultures were centrifuged and pellets were suspended and washed in the culture medium 2 times. After the final wash the bacterial pellet was diluted with culture medium 4×10^5^ times and 30 µL of this suspension was plated on LB/agar plates which were incubated at 37°C for 18 h. The number of colonies was counted and the results were expressed as a percentage of control cultures that were not treated with surfactant.

*** = *p*<0.001.

Taken together the results shown in Figures 1 and 2 and Table 1 clearly show that, in a CMC-dependent way, the C_n_TAB surfactants are bactericidal and the toxicity relative to the CMC of the surfactant decreases with increasing chain length. With regard to *E. coli* the toxicity ranking of all surfactants studied in this work, referring always to their respective CMCs as the reference concentration, was: C_10_TAB>C_12_TAB>C_14_TAB>SDS>DDPS>Triton X-100.

**Figure 3:**
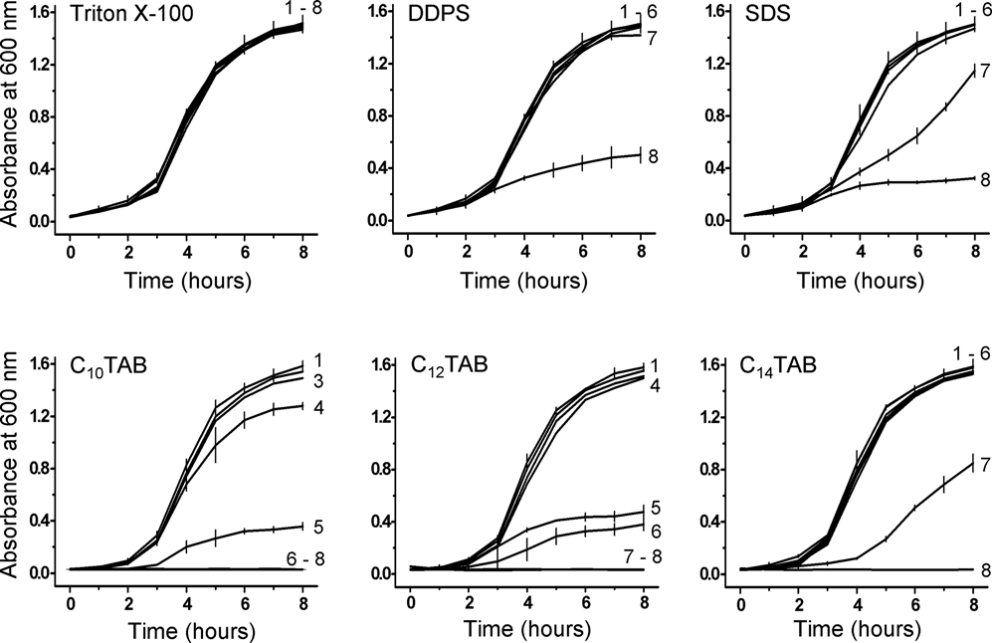
Effect of surfactants on the growth curves of *Pseudomonas aeruginosa*. Other details are as in Figure 1.

**Figure 4:**
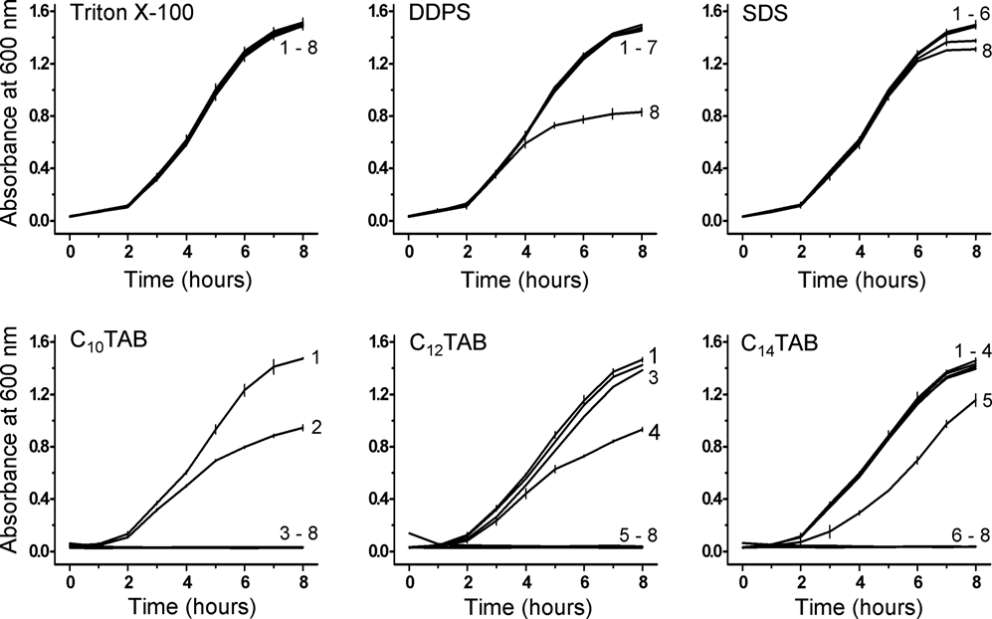
Effect of surfactants on the growth curves of Streptococcus agalactiae. Other details are as in Figure 1.

**Figure 5:**
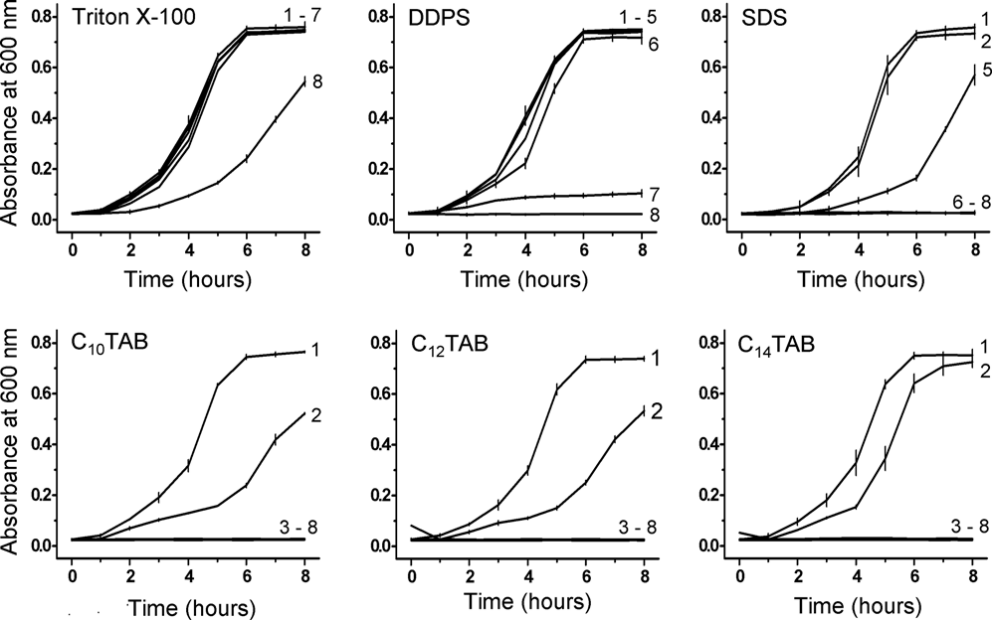
Effect of surfactants on the growth curves of *Enterococcus faecalis*. Other details are as in Figure 1.

In order to demonstrate the applicability of the above results to other bacteria involved in STD or urinogenital tract (related) infections we examined the toxicity of the same surfactants towards *Pseudomonas aeruginosa*, *Neisseria gonorrhoeae*, *Streptocococus agalactiae*, and *Enterococcus faecalis*. As seen in Figure 3, the results with *P. aeruginosa* were qualitatively similar to those with *E. coli* although the cationic C_n_TAB surfactants were slightly less toxic towards *P. aeruginosa* than they were towards *E. coli*. In the case of *N. gonorrhoeae*, the fastidious nature of this organism in culture did not allow us to perform experiments in homogeneous media. This organism was, therefore, cultured on solid media and surfactant toxicity was evaluated by subjecting the organism for 60 min to the different surfactants at different concentrations before plating the cultures. The results are shown in Table 2. *N. gonorrhoeae* shows the same pattern of susceptibility as the other Gram-negative bacteria examined, namely, weak susceptibility to Triton X-100, slightly higher susceptibilities to DDPS and SDS and very high susceptibility to the cationic C_n_TAB surfactants. In fact both C_10_TAB and C_12_TAB at concentrations of CMC/100, already reduce the number of viable organisms to about 50% after an exposure of one hour. In the case of *S. agalactiae* (Figure 4) the surfactant toxicities were qualitatively comparable to what was observed with *E. coli*, the cationic surfactants being slightly more effective in quantitative terms. *E. faecalis* (Figure 5) was, similar to *N. gonorrhoeae*, considerably more susceptible than the other three bacteria discussed. Triton X-100 was partly inhibitory to the growth of this organism at CMC/1, DDPS was almost totally inhibitory at CMC/2 and inhibitory effects of SDS were noticeable at CMC/10. This organism was extremely sensitive to all three cationic surfactants tested, growth inhibition being evident at concentrations of CMC/100.

**Table 2:** Effect of Surfactants upon the Viability of *Neisseria gonorrhoeae*.

Surfactant	Number of Colonies (as % of Control) after Exposure Time of 60 minutes to the Concentrations						
	CMC/100	CMC/50	CMC/25	CMC/10	CMC/5	CMC/2	CMC/1
Triton X-100	97.3±2.8	n.t.	n.t.	98.2±3.0	90.2±4.2	81.9±8.4	0 ***
DDPS	88.8±6.6	n.t.	n.t.	80.0±7.7 ***	36.3±5.2 ***	0 ***	0 ***
SDS	89.3±6.9	n.t.	n.t.	80.9±6.3 ***	0 ***	0 ***	0 ***
C_10_TAB	59.8±6.2 ***	12.1±4.0 ***	0 ***	0 ***	0 ***	0 ***	0 ***
C_12_TAB	55.5±9.1 ***	23.9±2.5 ***	0 ***	0 ***	0 ***	0 ***	0 ***
C_14_TAB	84.3±9.9	75.3±10.7	17.1±9.8 ***	0 ***	0 ***	0 ***	0 ***

n.t. = not tested.

*** = *p*<0.001.

### Fungicidal Properties of Surfactants

We chose the yeast *Candida albicans* as a fungal model to test for toxicity of the surfactants examined in this work. This organism is responsible for vulvovaginal candidiasis, a common mucosal infection in women of childbearing age. Furthermore, fungal infections have recently emerged as a growing threat to human health in seriously debilitated and immunocompromised diseases, such as AIDS and cancer [17].

**Figure 6:**
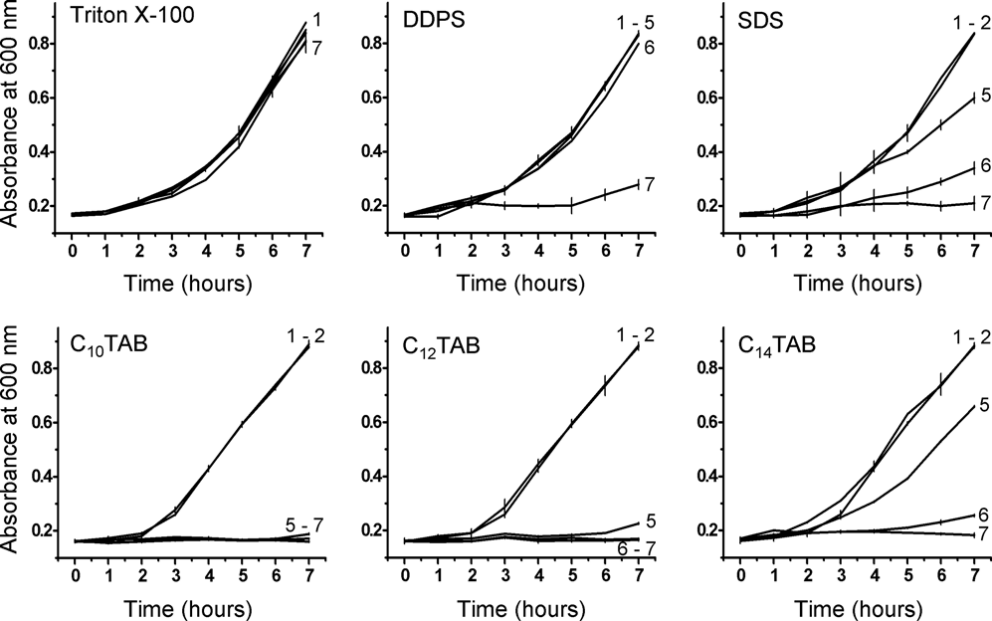
Effects of surfactants on the growth of *Candida albicans*. Other details are as in Figure 1.

Figure 6 shows the effect of all the surfactants examined upon growth curves of *C. albicans*. Although here, as in the case of *E. coli*, the toxic ranking of the surfactants examined, using the concentration criterion mentioned above, is C_10_TAB≈C_12_TAB>C_14_TAB>DDPS>SDS>Triton X-100, there are subtle differences that merit attention: *C. albicans* was more susceptible to the cytotoxic effects of SDS and DDPS than was *E. coli*. These differences suggest that surfactant toxicity is not just dependent upon the chemical structure of the surfactants but also upon the nature of the cell membrane, the *C. albicans* membrane being different both in chemical composition and physical properties from the membrane(s) of bacteria. Unlike the case of the bacteria, the toxicity of the C_n_TABs toward *C. albicans* is not related to the chain length.

### Surfactant toxicity towards polarized mammalian columnar epithelia (MDCK cells)

We chose Madine-Derby Canine Kidney (MDCK) cells as a model *in vitro* epithelial cell system. The reason for this choice was that: 1) This cell line is derived from a columnar epithelium and the vaginal columnar epithelium was shown to be the primary site of damage in studies of surfactant use in animal models [18]; 2) MDCK cells can be grown to a completely polarized state and there is a vast literature on the nature and properties of this polarized epithelial cell line in culture as we have used it; 3) In culture MDCK cells can be grown to confluence with relatively non-leaky tight junctions.

We studied the toxicity of the surfactants used in the cases of the microorganisms as described above towards the MDCK cells and, in addition, studied the toxicity of N-9 as well since this surfactant is widely used as a spermicide in condoms and was the object of unsuccessful Phase III studies [2], [3]. Toxicity towards polarized MDCK cells was measured by the MTT test (Table 3) and the Lactate Dehydrogenase (LDH) release test (Table 4). The MTT test is based on the reduction of MTT to form a formazan by mitochondrial and/or cytoplasmic dehydrogenases. The results showed that 16–18 h post-addition, the surfactants exhibited different degrees of toxicity. However, all surfactants tested exhibited CMC-dependent differences in cytotoxicity i.e. all were increasingly cytotoxic with increasing concentration relative to their respective CMCs. For Triton X-100 and DDPS, cytotoxicity was not observed up to a concentration of CMC/2, but it increased thereafter. N-9 was slightly less toxic to MDCK cells than Triton X-100. Among the C_n_TAB, C_14_TAB was the least toxic, the toxicity beginning to be noticeable at a concentration higher than CMC/5. In contrast C_10_TAB and C_12_TAB were noticeably toxic already at concentrations above CMC/25 (result not shown). SDS was toxic to the polarized MDCK cells at concentrations of CMC/5. The results with the MTT test for viability (Table 3) are well correlated with the observed release of LDH by the cells (Table 4). At concentrations higher than CMC/5, SDS caused the polarized epithelial cells to detach from the plastic surface resulting in an unreliable quantification of LDH release into the culture medium. The toxicity ranking of the surfactants studied towards the polarized epithelium model, using the concentration criterion mentioned above, was: C_12_TAB>C_10_TAB>SDS>C_14_TAB>Triton X-100>DDPS≥N-9. It is interesting to note that for the homologous series of cationic surfactants examined the toxicity to MDCK cells was not dependent upon the surfactant chain length in contrast to what was observed in the case of bacteria. Also, surfactant toxicity towards the mammalian cells is different, both qualitatively and quantitatively, from the toxicity that these same surfactants exhibited towards the bacterial and the fungal models. We suggest that this may have to do with the differences in physico-chemical properties of the cell membranes, the putative loci of interaction of surfactants with cells. Alternatively, surfactant toxicity towards cells may involve interactions that are not limited to the membranes alone.

**Table 3:** Effect of Surfactants on the Viability of Polarized and Confluent MDCK Cells as Evaluated by the MTT Test.

Surfactant	CMC/100	CMC/10	CMC/5	CMC/2	CMC/1
Triton X-100	93±9	92±10	88±11	84±5	33±11 ***
N-9	101±6	92±5	90±1	90±1	89±2
DDPS	97±7	86±1	84±1	84±8	62±6 ***
SDS	92±15	84±7 ***	68±6 ***	9±8 ***	8±8 ***
C_10_TAB	93±13	47±8 ***	5±7 ***	2±2 ***	1±2 ***
C_12_TAB	97±6	7±7 ***	2±7 ***	2±2 ***	1±2 ***
C_14_TAB	97±11	98±13	91±2	16±8 ***	2±2 ***

Cell viability is expressed relative to the viability of mock-exposed cells. Each value results from at least 3 independent experiments, in which each surfactant concentration was examined at least in triplicate. Data are shown as mean±SD (*n* = 3). ^***^, p<0.001.

**Table 4:** Effect of Surfactants on the Viability of Polarized and Confluent MDCK Cells as Evaluated by the LDH Leakage Assay.

Surfactant	CMC/100	CMC/10	CMC/5	CMC/2	CMC/1
Triton X-100	6±3	3±1	5±4	7±5	86±14 ***
N-9	3±1	3±2	5±3	3±2	22±6
DDPS	5±2	7±4	8±6	4±3	14±5
SDS	6±3	16±3	n.d.	n.d.	n.d.
C_10_TAB	7±1	53±7 ***	73±10 ***	86±3 ***	96±8 ***
C_12_TAB	7±3	87±9 ***	94±6 ***	94±5 ***	97±5 ***
C_14_TAB	6±1	6±1	15±7	86±14 ***	88±12 ***

Cell viability is expressed relative to the viability of mock-exposed cells. Each value results from at least 3 independent experiments, in which each surfactant concentration was examined at least in triplicate. Data are shown as mean±SD (*n* = 3). ^***^, p<0.001. In the case of SDS a significant lifting-off of the MDCK cells was observed at higher surfactant concentrations. In these samples the results of the LDH leakage test were considered to be not reliable and the values were, therefore, not determined (n.d.).

### None of the tested surfactants was able to prevent viral infection

**Figure 7:**
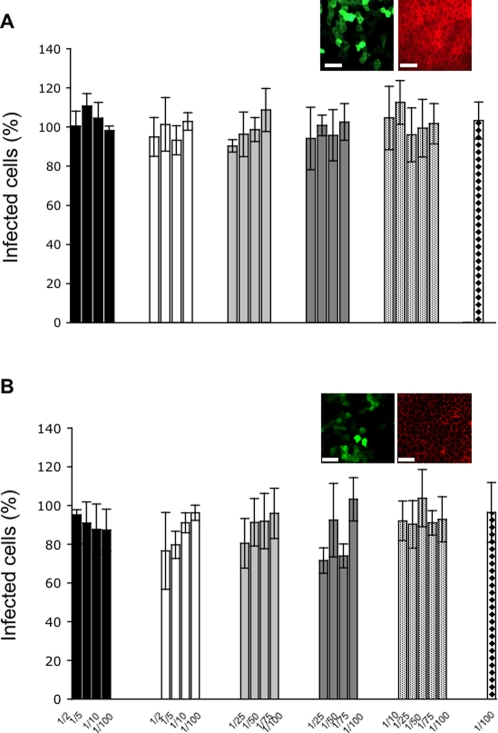
Effect of surfactants on adenoviral and lentiviral infections. Polarized MDCK cells grown on cover slips or polycarbonate filters were exposed to the adenovirus or lentivirus after the addition of the surfactants for about 1 h after which the cells were washed and placed in a fresh detergent- and virus-free culture medium. Infectivity was assessed 16–18 h or 48 h after virus and surfactant removal. A, MDCK infected with adenovirus expressing the GFP-GL-GPI. B, MDCK infected with lentivirus expressing GFP. The efficiency of infection was assessed by counting the number of infected cells (green cells, insert pictures) relatively to the total number of cells (given by staining the cells with rhodamine-phalloidin that labels the actin-cytoskeleton (red cells, insert pictures). Triton X-100, black columns. DDPS, empty columns. C_10_TAB, light grey columns. C_12_TAB, dark grey columns. C_14_TAB, columns filled with dots. SDS, columns filled with diamonds. Concentrations of the surfactants relative to their respective CMC are indicated at the graph abcissa of B. Infectivity is expressed as a percentage relative to cells infected in the absence of surfactant. Results are shown as mean values±standard deviations of 3–4 independent experiments in which each concentration was tested in duplicate.

Microbicides designed to protect against STD pathogens must do so without causing unacceptable toxic effects towards the vaginal epithelial cells. Indeed, the protective agents used would ideally be non-toxic towards the epithelial cells but highly toxic to the agents of infection. In practice, this ideal goal is difficult to attain and one must settle for a preferred toxicity towards the agent of infection, i.e. the toxic dose towards the agent of infection must be significantly lower than the toxic dose towards the cells or tissues that they invade. Viral infection of the vaginal epithelium is one of the most significant problems in STDs. We, therefore, tested the effect of surfactants on the ability of viruses to infect polarized MDCK cells. Adenovirus (a non-enveloped virus) and lentivirus (an enveloped retrovirus) were chosen as viral models since they are easily and relatively safely handled in the laboratory (both viruses are not able to replicate in humans). The lentivirus used here is derived from HIV-1 and pseudotyped with the envelope G-protein from the Vesicular Stomatitis virus. This confers higher physical stability [19] and broader tropism to the virus, which is able to infect a broader range of cell types. Both viral models were engineered to express a Green Fluorescent Protein (GFP) which facilitates microscopic inspection and analyses of infection. The surfactant concentrations used were, in all cases, inferior to those that had been previously judged to be toxic to the MDCK cells. The results (Figure 7), showed that in all cases there were no sub-toxic concentrations of the surfactants (with respect to MDCK cells) that could inhibit viral infection. It must be noted, however, that no surfactant promoted viral infection, comparable levels of infection being obtained in all cases.

### None of the surfactants tested was spermicidal at concentrations sub-toxic to MDCK cells

The effect of these surfactants on sperm viability, on sperm-membrane integrity as monitored by the Hypo-Osmotic Swelling (HOS) test, and as potential spermicides using the Sander-Cramer criteria were also assayed in human samples (Tables 5 through [Table pone-0002913-t006] 7). N-9, a nonionic surfactant, widely used as spermicide with demonstrable *in vitro* toxicity to a number of species of bacteria and viruses, including HIV, [4] was used as control. The effect of the surfactants on human sperm viability was measured by fluorescence after addition of SYBR-14 and propidium iodide. The sperm cells were incubated for 1 or 24 h with different surfactants and the results are summarized in Table 5. Except for Triton X-100, sperm viability decreased significantly when incubated with the surfactants, as compared with control. The viable sperm count declined with time and was CMC-dependent. Among the surfactants tested C_10_TAB and C_12_TAB were the most toxic. The toxicity ranking of surfactants studied towards human sperm cells, using the same concentration criterion as used before, was: C_10_TAB≈C_12_TAB>SDS>C_14_TAB>DDPS>Triton X-100. The results indicated again that the toxic effects of surfactants depend on the target cell. Cytotoxicity studies were also performed with N-9 and revealed that this spermicide exhibited toxicity towards human sperm at concentrations of CMC/1 (results not shown).

**Table 5:** Effect of surfactants, at 1 and 24 h post-exposure, on human sperm viability.

Surfactant	Exposure time (h)	CMC/100	CMC/10	CMC/5	CMC/2
Triton X-100	1	101.7±12.2	92.6±13.7	100.0±15.0	101.0±11.3
	24	101.2±10.8	84.3±14.2	101.8±17.8	75.3±25.7
DDPS	1	99.0±10.3	95.4±13.5	83.4±19.1	75.2±19.9
	24	94.5±10.9	77.0±22.5	56.9±23.5	12.5±11.5 **
SDS	1	93.6±18.2	86.6±8.2	28.2±16.2	0.6±1.4 *
	24	88.8±23.2	57.0±22.8	13.2±16.3 *	0.0±0.0 *
C_10_TAB	1	86.7±13.7	47.0±14.8	3.6±3.4 *	0.0±0.0 *
	24	76.1±13.1	7.1±6.0 **	0.0±0.0 **	0.0±0.0 **
C_12_TAB	1	92.2±11.3	48.6±25.7	6.1±4.7 **	0.0±0.0 **
	24	81.8±16.3 *	0.9±1.2 **	0.0±0.0 **	0.0±0.0 **
C_14_TAB	1	92.6±8.7	93.8±12.5	89.4±8.6	39.5±11.7 ***
	24	94.0±14.2	70.6±16.8	25.8±8.7 ***	6.3±11.1 ***

Cell viability was quantified as described in Material and Methods section and the results (average±SD) are shown in terms of % of control. ^*^, p<0.05; ^**^p<0.01; ^***^p<0.001.

**Table 6 pone-0002913-t006:** Effect of surfactants on the physiological integrity of human sperm cell membranes after the addition of hypo-osmotic buffer (HOS test).

	Triton X-100	N-9	DDPS	SDS	C_10_TAB	C_12_AB	C_14_TAB
CMC/100	94.4±7.3	81.8±25.9	41.1±8.3	90.4±6.5	82.1±22.0	72.5±19.4	79.4±6.9
CMC/2	91.4±9.4	69.7±18.9	7.2±3.4 *	22.1±15.7 **	15.0±8.3 *	5.8±5.6 ***	36.2±22.4

Results (average±SD) are shown as the percentage of intact cells relative to the control. ^*^, p<0.05; ^**^p<0.01; ^***^p<0.001.

**Table 7:** Effect of surfactants on the sperm motility assessed by the Sander-Cramer assay.

	Triton X-100	N-9	DDPS	SDS	C_10_TAB	C_12_TAB	C_14_TAB
CMC/2	−	−	−	−	−	+	−
CMC×1	−	−	−	+	+	+	−
CMC×5	+	+	+	+	+	+	+
CMC×10	+	+	+	+	+	+	+

The percentage of motile sperm was determined microscopically as described and a plus sign means that 100% spermatozoa were immobilized within 20 seconds of exposure to the surfactants.

The viability results were complemented with the HOS test, an approach that serves in early detection of structural and/or functional alterations of sperm membranes. The results of the HOS test obtained 1 h post-addition of the surfactants showed that at CMC/2 all surfactants except Triton X-100, C_14_TAB and N-9 were able to affect the integrity of the plasma membrane of the sperm cells (Table 6). These results were CMC dependent and in the case of SDS, C_10_TAB, C_12_TAB were well correlated with the viability results. In contrast, with DDPS we detected no correlation between the HOS assay and sperm viability.

Generally compounds that fail to immobilize sperm within 20 s are unacceptable because of the rapidity with which sperm migrate into the cervix and upper reproductive tract. Hence the Sander-Cramer test was used to identify the minimum concentration of surfactant, relative to its CMC, required to completely immobilize spermatozoa within 20 s. C_12_TAB was highly effective (Table 7), causing complete immobilization of spermatozoa within the allowed 20 s at concentrations of CMC/2. C_10_TAB and SDS were effective at CMC/1 and DDPS, C_14_TAB and N-9 at CMC×5. At these spermicidal concentrations all surfactants were shown to be extremely toxic to the mammalian columnar epithelial cells (Table 3 and 4) and N-9 exhibited toxicity towards MDCK cells at concentrations of CMC×2 (result not shown). Furthermore, these spermicidal concentrations were also higher than the minimum inhibitory concentrations (CMC/10-CMC/100) required to inhibit the growth of bacteria and fungi.

## Discussion

In this work we have demonstrated an easy and fast *in vitro* methodology to screen candidate microbicide and spermicide surfactants in the struggle against conception and STD. This methodology demonstrated that the quaternary C_n_TAB surfactants, at non-toxic concentrations towards polarized epithelial cells, are effective against bacteria and yeast, but are not spermicidal nor do they offer protection against adenovirus and lentivirus infection at concentrations that are sub-lethal to epithelial cells. We have also shown that several other surfactants (non-ionic, zwitterionic, and anionic) tested by us are neither spermicidal nor bactericidal, nor protective against viral infection of polarized epithelial cells, below concentrations at which they are toxic to the epithelial cells themselves. This result was somehow unexpected for SDS (the anionic surfactant tested by us), which is considered an attractive candidate microbicide due to its lower cytotoxicity [4], [13], and for N-9 which is commonly used, albeit at very high concentrations, as a contraceptive in condoms. Anti-bacterial and anti-fungal compounds, with potential to be used in female-controlled vaginal products, are of particular interest for two reasons: 1) Consumer preference studies suggest that most women worldwide prefer vaginal microbicides to condoms; 2) There is an urgent need for cheap and safe new microbicides that could be used in poor regions around the world. Surfactants, if effective, would be ideal candidates since they are cheap and easily applied and also have reasonably long shelf lives. However, it appears from our present results, that considerably more work is required to understand exactly how surfactants kill cells (of whatever type) and to design surfactants that will be able to fulfill specific and well-understood tasks.

We may highlight two interesting observations in the present study: 1) There appears to be some selectivity in the action of the cationic quaternary ammonium compounds on bacteria and yeast on the one hand and mammalian epithelial cells and spermatozoa on the other. C_10_TAB does not show cytotoxicity towards mammalian cells at concentrations between CMC/25 and CMC/100 but is toxic to all the bacteria (with the exception of *P. aeruginosa*) and to *C. albicans* at concentrations of CMC/25 or lower. It also has a rapid microbicidal action (<30 min). 2) The anti-bacterial and anti-fungal activities were related to the surfactant CMC. The chain length dependence (C_10_TAB>C_12_TAB>C_14_TAB) may be related to the fact that the absolute concentrations are also an important determinant in their microbicidal action since the CMC of C_10_TAB is about 10-fold higher than that of C_12_TAB and about 100-fold higher than that of C_14_TAB. Similar results have been reported for amphiphilic betaine esters with a chain length of C_10_–C_14_ [14]. It must, however, be emphasized that this correlation is not observed when the targets are MDCK cells or spermatozoa where C_12_TAB is more toxic than C_10_TAB or C_14_TAB.

Though the disinfectant properties of the cationic surfactants have been extensively reported, their mechanism of action is not fully understood. It has, plausibly, been proposed to involve changes in the surface electrostatic potential of the membranes into which the hydrophobic portion of these surfactants is inserted as a consequence of the hydrophobic effect [20]. Their action on bacterial and fungal cells probably does not involve disassembly of the membranes since the concentrations at which toxicity is observed are well below the CMC. A possible mechanism of action that has been only infrequently considered in the literature is the effect of surfactants in the cytoplasm of the cells to which they are toxic [21]. The insertion of surfactants with a single hydrophobic chain into membranes occurs at a rate that is almost diffusion controlled and is, therefore, an extremely rapid process. After insertion, the surfactant can translocate across the membrane and establish equilibrium between the extracellular space and the cytoplasm. The insertion and translocation processes together have a half-time of about 20 min (see, for example, [22]) which is compatible with the times we observe for toxicity towards *E. coli*. Once in the cytoplasm the surfactant may interact with several cytoplasmic components and alter their activity. In particular, the cationic surfactants may be expected to interact with negatively charged polymers such as DNA and RNA for which they are known to have very high affinities and with which they form compact structures [23]. In fact, sequestration of the cationic surfactants by interaction with nucleic acid polymers within the cell would accelerate attaining surfactant-concentration equilibrium across the cell membrane. The results reported here suggest that quaternary ammonium surfactants interact with different cells differently which may well imply having different cellular targets with different susceptibilities in the different cell models. These dissimilar mechanisms of action may be related to the differences in lipid and protein content between the cellular plasma membranes or to the manner in which DNA and RNA is protected within the cell models we used. We are investigating this matter further.

In conclusion, although animal models and pre-clinical studies offer more complete experimental conditions, *in vitro* experiments have the advantage of convenience, flexibility, speed, and low cost. The results obtained from the present systematic study provide insights on the applicability and the safety of using surfactants as contraceptive, anti-viral, and anti-microbial agents. On the basis of these results, none of the surfactants tested by us could qualify for testing with animal, and much less with human, subjects.

## Materials and Methods

### Reagents

MDCK cell culture media were from Invitrogen or PAO Laboratories. All chemicals were of the highest commercially available purity and were used as received. 3-(4,5-Dimethylthiazo-2-yl)-2,5-diphenyltetrazolium bromide (MTT), the nonionic surfactant Triton X-100 (TX-100), the anionic sodium dodecyl sulfate (SDS), the zwitterionic (N-dodecyl-N,N-dimethylammonium-propanesulfonate, DDPS) and the cationic surfactants: dodecyltrimethylammonium bromide (C_12_TAB), and tetradecyltrimethylammonium bromide (C_14_TAB) were obtained from Sigma. Decyltrimethylammonium bromide (C_10_TAB), was from Fluka. Stock solutions of surfactants were prepared as multiples of their CMC [24]. Rhodamine-phalloidin and the LIVE/DEAD® Sperm Viability Kit (L-7011) were purchased from Molecular Probes (Eugene, OR, USA). The kit contains two DNA-binding fluorescent stains: SYBR-14 and propidium iodide. Yeast extract and Bacto-Peptone were from DIFCO and LB from Invitrogen. Bacterial agar was from OXOID, UK.

### Culture of Bacteria and C. albicans

Wild type *E. coli* and *Pseudomonas aeruginosa* were isolated from human necropsies. *Enterococcus faecalis* were isolated from human urinary tissues. *Streptococcus agalactiae* (138813) and *Neisseria gonorrhoeae* (49226) were from the ATCC. *E. coli*, *P. aeruginosa*, *S. agalactiae* and *E. faecalis* were grown overnight in Luria broth medium at 37°C and shaking at 200 rpm. Bacterial and yeast growth was assessed by measuring the absorbance at 600 nm as a function of time, in presence or absence of detergents.

Bactericide activity was quantified by plating bacteria that had been previously incubated with surfactants on LB/agar plates after separation from the added surfactants by centrifugation. The LB/agar plates were then incubated at 37°C for 16–18 h and microbicide efficiency was determined by counting the number of colonies formed.

*N. gonorrhoeae* was grown on Chocolate Agar PolyViteX® plates (BioMerieux), and GCB plus Kellogg's supplement [28] broth was used for the surfactant assays. In these assays, a bacterial suspension was made by using a 2 McFarland standard. From this suspension, 10 µL were used as inoculum in 1 mL of each surfactant concentration tested. Controls containing the broth were tested concurrently. The mixture was incubated at 37°C in a 5% CO_2_ atmosphere for 1 h. The samples were then diluted 100-fold and 100 µL were inoculated on Chocolate Agar plates. The plates were incubated at 37°C in a 5% CO_2_ atmosphere for 24 h and scored for colony growth. Counts from bacteria treated with surfactants were compared with those from the controls which contained no surfactant.

*C. albicans* was grown in conical flasks in YEPD medium (1% Yeast Extract, 2% Bacto-Peptone and 2% Glucose) on an orbital shaker at 37°C and shaking at 200 rpm.

### MDCK cells, their culture and virus infection

MDCK cells were grown on glass-coverslips or on 12-mm Transwell polycarbonate filters (Corning Costar, Corning, NY) for 4 days in MEM with 10% FCS. On the third day, the cells were infected with adenoviruses or lentiviruses for 1 hour and the surfactants were added 10 min before the infection. After incubation for 1 h the viruses and surfactant were removed and fresh medium was added to the cells. Only in the experiments with lentivirus the infection was done in the presence of 4 µg/mL polybrene (Sigma). At 16–18 h (adenovirus) or 48 h (lentivirus) post-infection, the cells were fixed with PFA, permeabilized with Triton X-100 and incubated with rhodamine-phalloidin. The glass-coverslips or the polycarbonate filters were then mounted and observed under a Zeiss Axioskop 2 plus or a Zeiss confocal microscope.

### Generation of recombinant adeno- and lentiviruses

The generation of the plasmid (GFP-GL-GPI) and adenovirus used were described previously [25]. Lentivirus encoding GFP were produced as described elsewhere [26].

### Evaluation of in vitro toxicity of surfactants towards mammalian cells

Viable cells were measured by their ability to reduce the tetrazolium salt, 3-(4,5-dimethylthiazole-2-y)-2,5-diphenyltetrasodiumbromide (MTT), to a formazan dye detectable by spectrophotometric analysis in a microplate reader.

The activity of the cytoplasmic enzyme LDH in the extracellular medium, which evaluates plasma membrane integrity was measured spectrophotometrically according to the method described by Bergmeyer and Bernt [27] by following the rate of conversion of reduced nicotinamide adenine dinucleotide (NADH) to oxidized nicotinamide adenine dinucleotide (NAD^+^), at 340 nm.

### Spermicidal activity of surfactants

The effect of surfactants on human sperm was quantified using three distinct assays for sperm viability, sperm integrity and spermicidal activity of added substances.

Human sperm was obtained from the Human Reproduction Laboratory, Department of Maternal-Fetal Medicine, Genetics and Human Reproduction (Coimbra University Hospitals), from healthy males undergoing routine semen analysis or fertility treatment. All donors signed informed consent forms, and all human material was used in accordance with the appropriate ethical guidelines provided by the Coimbra University Hospitals and its Internal Review Board (IRB). Fresh semen samples were obtained by masturbation after 3–5 days of sexual abstinence, and routine seminal analysis was performed according to the World Health Organization guidelines (WHO, 1999). [29]. Only normal sperm samples were used.

The effect of surfactants on sperm viability was determined microscopically after nuclear staining with SYBR-14 and propidium iodide (PI), both prepared in DMSO [30]. After a 1 or 24 h exposure to different dilutions of surfactants samples were then mounted in a microscope slide and immediately observed under epifluorescence (Zeiss Axioplan 2, Carl Zeiss, Göttingen, Germany). A total of 200 sperm were counted and analysed per sample, and the results expressed in terms of viable (PI-negative) cells.

The Hypo-Osmotic Swelling (HOS) test was carried out 1 h after the addition of surfactants. Briefly, after treatment with surfactants human sperm was incubated in a 150 mOsm hypo-osmotic saline solution at 37°C during 60 min [31]. Then the mixture was examined immediately under a phase-contrast microscope. Sperm cells are very rigid with a limited amount of cytoplasm, and therefore a reaction to hypo-osmotic medium results, not in visible “swelling” (as with other cell types), but in the coiling of the sperm tail. Thus, sperm with intact and functional membranes present typical coiled tails, contrasting with damaged sperm, were no morphological alterations are detected and straight tails are the norm. Results higher than 60% of coiled tails (i.e. sperm with intact membranes) are considered within the normal range.

Activity of surfactants on sperm motility was determined by the Sander–Cramer assay [32]. Briefly, spermatozoa were placed in PBS with glucose and BSA containing appropriate dilutions of the surfactants. After vortexing for 10 s, samples were placed on slides and ten fields were rapidly examined under high-power magnification with a phase-contrast microscope. In each field a total of 100 spermatozoa were counted. According to this test, a result is scored as positive if 100% spermatozoa became completely immotile within 20 s.

### Statistical analysis

Results were expressed as the means±standard deviations (S.D.). Statistical significance was assessed by the ANOVA test. A *p* value of <0.05 was considered to be statistically significant.
